# Structural insights into the Urm1-Uba4 pathway and its biological roles

**DOI:** 10.1042/EBC20253041

**Published:** 2026-02-13

**Authors:** Dominika Kwasna, Keerthiraju E. Ravichandran, Anna Biela, Sebastian Glatt

**Affiliations:** 1 Malopolska Centre of Biotechnology (MCB), Jagiellonian University, Krakow, Poland; 2 Postgraduate School of Molecular Medicine, Warsaw, Poland; 3 Department for Biological Sciences and Pathobiology, University of Veterinary Medicine Vienna, Vienna, Austria

**Keywords:** ubiquitins, ubiquitin signaling, TRNA, persulfidation, thiolation, Urm1

## Abstract

Ubiquitin-related modifier 1 (Urm1) is a unique and evolutionarily conserved member of the ubiquitin-like protein (UBL) family that represents a molecular link between ancestral sulfur carrier proteins (SCPs) and canonical eukaryotic UBLs. Urm1 is required for the thiolation of tRNAs and a non-canonical post-translational modification, called ‘urmylation’. Activation of Urm1 by its E1-like enzyme, ubiquitin-like protein activator 4 (Uba4), involves the sequential adenylation, thioesterification, and thiocarboxylation of Urm1’s C-terminus. Thereby, Urm1 can provide sulfur for the tRNA modification reaction or catalyze its conjugation to target proteins through a mechanism that is independent of E2-conjugating enzymes and E3 ligases. Recent structural studies have resolved several key intermediates of the fungal Uba4-Urm1 system, shedding light onto its two distinct subdomains and their dynamical interplay. Notably, Urm1 also interacts with several additional up- or downstream partners of the two pathways. Foremost, urmylation couples an UBL-conjugation reaction with the persulfidation of a cysteine residue in the target proteins. This protective oxidative post-translational modification underscores Urm1’s central role in redox regulation and cellular stress responses. Here, we aim to summarize the most recent mechanistic insights and structural advances in the eukaryotic Urm1-Uba4 pathway.

## Introduction

Ubiquitin-related modifier 1 (Urm1) is a distinctive and evolutionarily speaking ancient member of the ubiquitin-like protein (UBL) family that is involved in a variety of cellular functions in eukaryotes. Unlike conventional UBLs, Urm1 exhibits two distinct functionalities: (i) Urm1 acts as a sulfur carrier protein (SCP) crucial for the thiolation of uridines in the tRNA anticodon and (ii) participates in the post-translational modification of cysteine-containing target proteins under oxidative stress [1–5]. Urm1 serves as a molecular link between prokaryotic SCPs and eukaryotic UBLs, highlighting its unique position in UBL evolution [3,6]. Urm1 is activated by an E1-activating enzyme, called ubiquitin-like protein activator 4 (Uba4). Like in other E1-UBL systems, the C-terminus of Urm1 is first adenylated in an ATP-dependent fashion and then forms a transient thioester with a conserved cysteine residue of Uba4. Whereas most UBLs subsequently transfer to an E2-conjugating enzyme, Uba4 uses its unique Rhodanese (RHD) domain to further thiocarboxylate the C-terminus of Urm1 [7]. Thiocarboxylated Urm1 can transfer the attached persulfide group to tRNAs via the Ncs2/Ncs6 thiotransferase complex [8] or to proteins via a noncanonical protein conjugation reaction [9]. The thiolation of U_34_ in tRNAs assures translation fidelity during protein synthesis [10–12], while the conjugation activity is linked to cellular responses to oxidative stress [1,2,13–16]. Furthermore, Urm1 catalyzes protein persulfidation during the conjugation reaction [9], a protective post-translational modification that shields cysteine residues from irreversible oxidation and modulates protein function and redox signaling [17]. This unique combination of functions underscores the distinct role of Urm1-mediated RNA- and protein modifications.

Finally, a study [18] revealed that Urm1 promotes condensate formation in yeast and human cells. By default, Urm1 seems to localize to nuclear and cytoplasmic condensates and target low-complexity sequences in proteins, which leads to their relocation, enrichment, phase separation, and formation of cellular condensates. Under stress conditions (e.g. heat shock or inhibition of translation), Urm1 is up-regulated, further enhancing urmylation and promoting phase separation, which appears to be essential for cellular fitness and stress resilience across eukaryotes [18]. This review aims to highlight recent insights into the Uba4-Urm1 system, including structures, activation mechanisms, and the principles of its unique Ubiquitin-like protein conjugation reaction.

### Two routes for Urm1 to mediate RNA- and protein modifications

**Figure 1:**
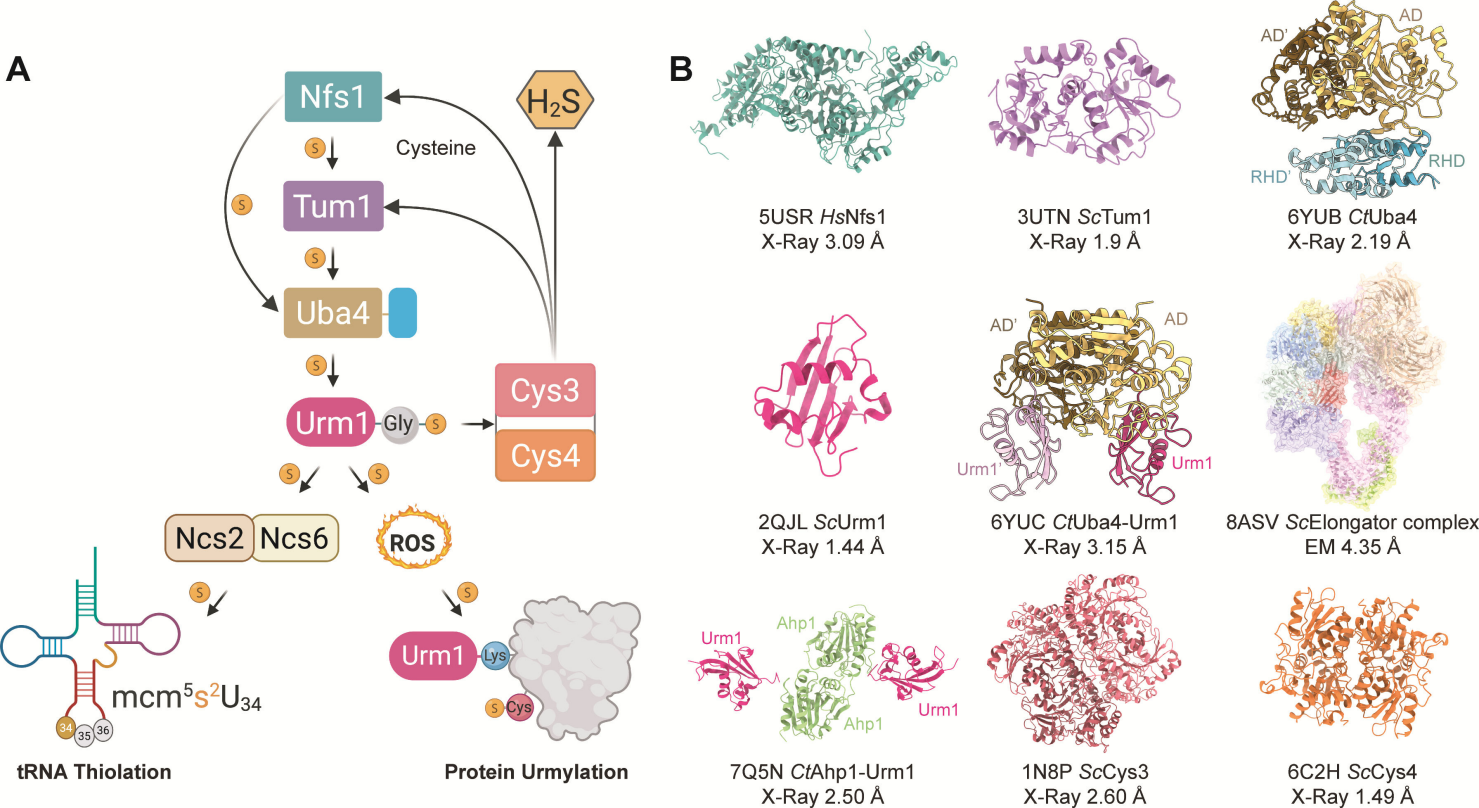
The dual role of Urm1 and its pathway.

The presence of modifications at the wobble position of tRNAs is critical for ensuring accurate translation and proper decoding of m^6^A-containing mRNAs [12], which affects transcriptome turnover as well as proteome stability. In eukaryotes, the modification activities of the Elongator pathway (5-methoxycarbonylmethyl; mcm^5^) and the subsequent thiolation cascade complete the formation of 5-methoxycarbonylmethyl-2-thiouridine (mcm^5^s^2^U_34_) [19]. The sulfur for the thiolation is sourced from free cysteine molecules via a conserved relay system that involves the cysteine desulfurase Nfs1, Thiouridine modification protein 1 (Tum1), and Ubiquitin-like protein activator 4 (Uba4). After the sulfur group has reached the C-terminus of Urm1, it is transferred via the Ncs2/Ncs6 complex (CTU1/CTU2 in humans) to tRNA^Lys^_UUU_, tRNA^Glu^_UUC_, and tRNA^Gln^_UUG_ in yeast, and additionally to tRNA^Arg^_UCU_ in vertebrates [20] (Figure 1A). In bacteria, a similar wobble base thiolation occurs through a different multi-protein cascade [21,22]. Despite their functional similarities, the eukaryotic sulfur relay system diverges significantly from the bacterial pathway, highlighting the functional importance of the U_34_ modification itself and the possible evolutionary adaptations in the family of tRNA modifying enzymes [5,22]. Tum1, together with cystathionine γ-lyase (Cys3) and cystathionine β-synthase (Cys4), constitutes key elements of the trans-sulfuration pathway in yeast and has been demonstrated to modulate intracellular H_2_S levels [23] (Figure 1A). Depletion, mutation, or removal of Cys3 or Cys4 also results in decreased tRNA modification levels, indicating a direct linkage between tRNA thiolation and the trans-sulfuration pathway [23,24].

(**A**) Schematic representation of the dual role of Urm1 in tRNA thiolation and protein urmylation. Illustration created with Biorender.com (**B**) Three-dimensional crystal and cryo-EM protein structures of the enzymes involved in Urm1 pathway. PDB IDs of respective proteins: *Hs*Nfs1-5USR [27]; *Sc*Tum1-3UTN [33]; *Ct*Uba4-6YUB [26]; *Sc*Urm1-2QJL [34]; *Ct*Uba4-Urm1-6YUC [26]; *Sc*Elongator Complex-8ASV [37]; *Ct*Ahp1-Urm1-7Q5N [9]; *Sc*Cys3-1N8P [35]; *Sc*Cys4-6C2H [36].

Initially, Urm1 and Uba4 were identified as components of a protein conjugation system in yeast [13]. Later, it was described that the Urm1-Uba4 axis primarily functions in tRNA thiolation as described above [4,6,8,25]. However, Urm1 was shown to conjugate to various target proteins in a Uba4-dependent process *in vivo* [13–16]. Alkyl hydroperoxide 1 (Ahp1), which functions as an antioxidant that mitigates oxidative damage, was identified as one of the main target proteins of Urm1 [16]. Chemical probing experiments revealed the necessity of the thiocarboxy group at the C-terminus of Urm1 for the isopeptide bond formation [14,25]. A surface lysine residue (Lys32) of Ahp1 was identified as the primary site of Urm1 attachment [2], but other proximal lysine residues on the surface of Ahp1 can also be linked to Urm1 [9]. The conjugation reaction is triggered exclusively under oxidative stress, it is influenced by oxidizing agents such as diamide and H_2_O_2_, and the conjugation patterns remain similar [9,14]. *In vitro* studies have demonstrated that Urm1 can also form covalent conjugates with Uba4 in the presence of oxidative agents, showing that the catalytic cysteine residue of Uba4 (Cys202) can form a stable isopeptide bond with Urm1 under stress conditions [26]. In summary, several studies substantiate the involvement of Urm1 in a ubiquitin-like urmylation reaction, in parallel to its canonical function as a sulfur donor for the tRNA thiolation cascade.

### Molecular and structural information of pathway components

The structural determination of enzymes involved in the Urm1 pathways has been instrumental in revealing the conformational transitions and intermediate states that underpin this conserved system (Figure 1B). The cysteine desulfurase Nfs1 is crucial for Fe-S cluster assembly and forms a functional homodimer in prokaryotes. In eukaryotes, the Nfs1 dimer is an integrated part of multi-subunit complexes in mitochondria with ISD11, ISCU, ACP, and FXN. The human cysteine desulfurase NFS1 was initially crystallized in complex with ISD11, an *E. coli* acyl-carrier protein (PDB: 5USR and 5WGB) [27,28]. Furthermore, crystal as well as single particle cryo-EM structures of different human NFS1 assemblies at different reaction intermediates have become available at high resolution (PDB: 5WLW, 5WKP, 6NZU, 6UXE, 6W1D, 6WI2, 7RTK, 8PKA, 8PK9, 8PK8, 8TVT, 8RMG, 8RMF, 8RME, 8RMD, 8RMC) [28–32]. The structure of the yeast tRNA-thiouridine modification protein 1 (*Sc*Tum1) was resolved at 1.9 Å and showed two rhodanese-like domains (RLDs), where only the C-terminal RLD contains a conserved cysteine residue required for sulfur transfer (PDB: 3UTN) [33]. The crystal structure of yeast Urm1 (*Sc*Urm1) alone was determined at 1.4 Å resolution (PDB: 2QJL) [34]. Urm1 forms a stable dimer through a combination of hydrophobic and polar interactions, primarily involving its C-terminal residues. It has been proposed that this dimerization may protect the highly reactive C-terminal glycine residues prior to its engagement with other partners and conjugation [34].

Crystal structures of the apo form of *Chaetomium thermophilum* Uba4 (PDB: 6YUB) [26] and its complex with Urm1 (PDB: 6YUC) [26] were resolved at 2.2 Å and 3.15 Å, respectively, and are discussed in greater detail elsewhere in this review. To date, the only known *in vivo* substrate of urmylation, Ahp1, has been crystallized in complex with *Ct*Urm1 at 2.5 Å resolution (PDB: 7Q5N) [9]. In addition, the crystal structures of yeast cystathionine γ-lyase (Cys3) and cystathionine β-synthase (Cys4) were obtained at 2.6 Å (PDB: 1N8P) [35] and 1.5 Å (PDB: 6C2H) [36], respectively. Finally, the yeast Elongator complex was resolved using single-particle cryo-electron microscopy at an overall resolution of 4.3 Å (PDB: 8ASV) [37] (Figure 1B).

### How Uba4 works and activates the C-terminus of Urm1

**Figure 2:**
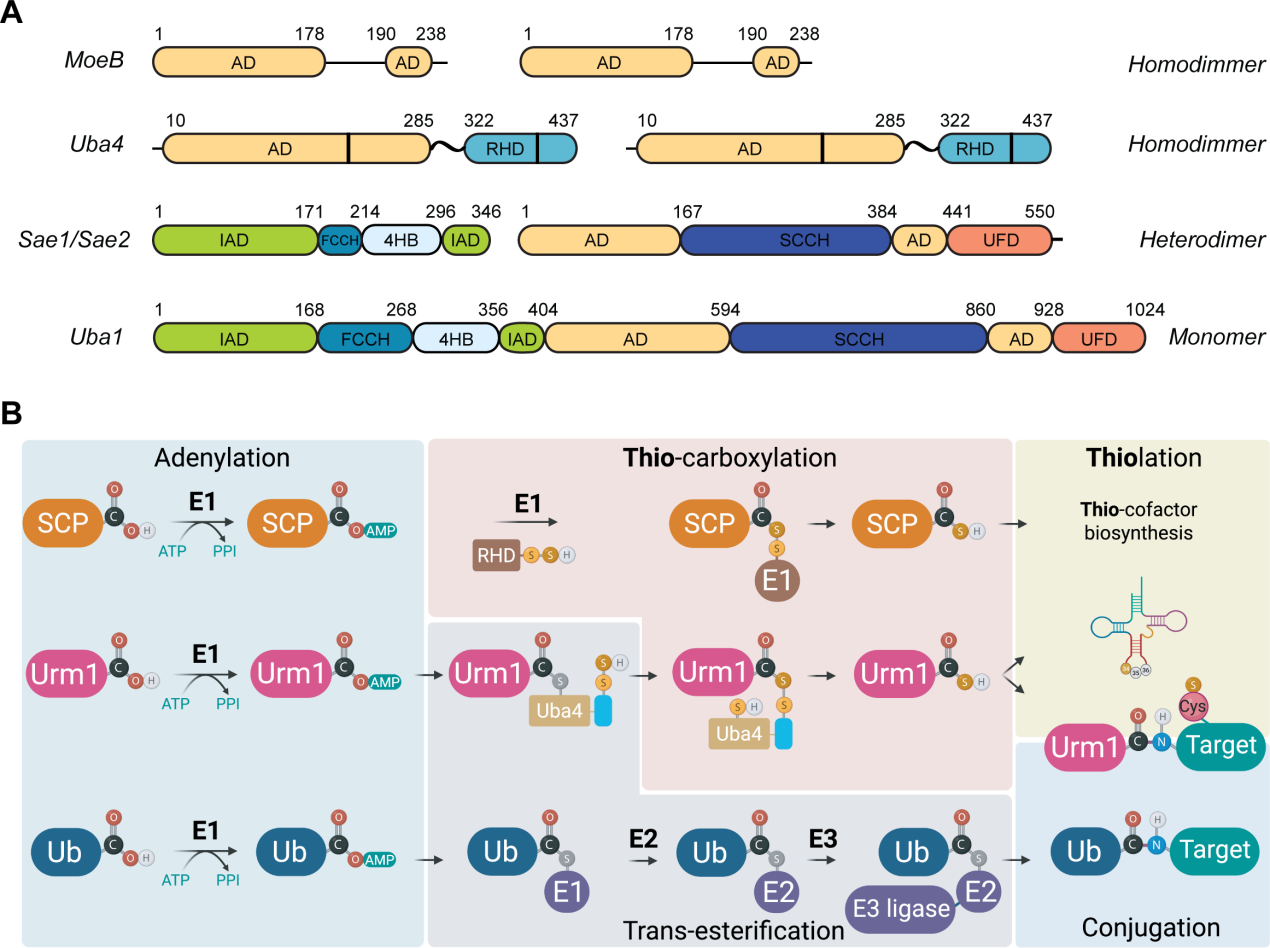
Domain overview of activating enzymes and mechanistical comparison of the SCP, Urm1, and Ubiquitin pathways.

 Yeast Urm1 is activated by its noncanonical E1 enzyme Uba4 (MOCS3 in humans), which contains an adenylation domain (AD) and a Rhodanese (RHD) domain, connected by a flexible linker. However, Uba4 lacks the ubiquitin-fold domain (UFD), which is typically found in E1-activating enzymes of other known UBL pathways [26] (Figure 2A). First, Urm1 is adenylated by the AD of Uba4, followed by the formation of a thioester intermediate between Urm1 and a conserved cysteine residue in the so-called ‘crossing loop’ of Uba4. Up to this point, Urm1 activation proceeds analogously to most other E1-UBL systems. Subsequently, the RHD domain of Uba4, which carries a persulfide group on its active site cysteine, transfers a sulfur atom to the C-terminus of Urm1. The process concludes with a reductive cleavage, yielding the thiocarboxylated Urm1 (Urm1-COSH) product (Figure 2B).

(**A**). Schematic domain representation of *E. coli* MoeB, *Ct*Uba4, *Hs*Sae1/2, and *Sc*Uba1. (**B**). Urm1: The Enigmatic Bridge Between Prokaryotic Sulfur Carriers and Eukaryotic UBLs. Prokaryotic sulfur carrier proteins (SCP) and eukaryotic Urm1 and ubiquitin require E1-type enzyme-mediated adenylation. In the ubiquitin pathway, E1 activation creates an E1~Ub thioester, which is transferred to E2 and E3 enzymes for lysine conjugation. In contrast, SCP adenylation lacks a thioester intermediate and involves a dedicated desulfurase and rhodanese domain (RHD) protein, facilitating sulfur transfer and thiocarboxylation. For Urm1, adenylation yields a thioester linked to Uba4’s adenylation domain, which then transfers to a persulfide on the RHD of Uba4. The RHD of Uba4 obtains its persulfide from either Nfs1 directly or Tum1 indirectly, with cysteine as the sulfur donor (not shown). Subsequently, reductive cleavage of the acyl-disulfide releases Urm1 thiocarboxylate (Urm1-COSH) for downstream tRNA thiolation via Ncs2-Ncs6 (not shown). Urm1-COSH is also essential for urmylation, leading to persulfidation and ubiquitin-like conjugation of proteins. Illustration created with Biorender.com.

Although the presence of those intermediates has been predicted [4,7,8,10,38], structural information on how these two domains orchestrate the individual steps during activation and thiocarboxylation of Urm1 remained elusive until recently [26,39]. Of note, ancestral UBLs in bacteria that are structurally related to Urm1 (e.g. ThiS and MoaD) function exclusively as SCPs, mediating sulfur transfer reactions without being involved in covalent protein modifications and without interacting with E2-conjugating enzymes and/or E3 ligases. Nonetheless, these prokaryotic SCPs are still activated by E1-like enzymes (e.g. ThiF and MoeB), which contain a single adenylation domain (AD). The respective E1 enzymes adenylate the C-terminus of the respective UBL, but neither forms a thioester intermediate with the UBL [21,22] nor contains an integrated RHD domain. In these pathways, separate RHD proteins (e.g. GlpE, TST, RhdA) thiocarboxylate the adenylated UBL to allow subsequent sulfur transfer reactions. Interestingly, it was recently shown that Tum1, a RHD protein, can partially rescue the thiocarboxylation of Urm1 and complement a nonfunctional RHD domain of Uba4 [39].

 The Uba4-Urm1 axis shows many functional similarities to both prokaryotic SCPs and eukaryotic UBL systems. These features shared between both worlds suggest that Urm1 may have served as an evolutionary precursor to the vast variety of eukaryotic UBL systems [6]. Furthermore, structural analyses have confirmed additional similarities between Uba4 and typical E1 enzymes that activate UBLs [26]. These structural and functional similarities again corroborate the theory that canonical E1-UBL systems originated from Urm1-like ancestors. As these pathways evolved, they maintained the initial activation steps until the thioester bond formation, they lost the thiocarboxylation step, while developing enhanced substrate specificity and regulatory complexity via the involvement of E2-conjugation enzymes and E3 ligases. Therefore, the Uba4-Urm1 system exemplifies an evolutionary transitional stage that connects primal prokaryotic sulfur-carrier systems to the advanced eukaryotic ubiquitin-like modification apparatus (Figure 2B).

### Structures of Uba4 and the Uba4-Urm1 complex

The crystal structure of full-length Uba4 was obtained by analyzing Uba4 from the thermophilic fungus *Chaetomium thermophilum* (also known as *Thermochaetoides thermophila*). This organism harbors a full fungal proteome, which has adapted to the high temperatures and exhibits enhanced protein stability, making it particularly suitable for structural biology, like X-ray crystallography [26]. Interestingly, the full-length Uba4 structure is arranged in an asymmetric fashion as a homodimeric complex of two Uba4 monomers with an asymmetric positioning of their ADs and RHDs, which individually form dimers on their own. This asymmetric assembly/architecture enables Uba4 to perform dual enzymatic functions, adenylation and sulfur transfer, which are essential for the thiocarboxylation of Urm1. Each of the two nucleotide-binding sites involves highly conserved residues from both monomers, suggesting that homodimerization of the ADs is essential for efficient Urm1 adenylation. Of note, the formation of a stable Uba4-Urm1 complex requires the simultaneous presence of Uba4, Urm1, and ATP, which is in contrast to other canonical and noncanonical E1 enzymes that can bind their respective UBL even in the absence of ATP and Mg²^+^ ions [40–43].

 Further insights into Urm1 activation by Uba4 were gained by trapping a thioester intermediate. In detail, the Uba4_C202K_ mutant was used, which forms a stable isopeptide bond between the activated C‐terminus of Urm1 and the ε‐amino group of the artificially introduced lysine residue. This strategy has previously been used to trap the E2-Ubiquitin complex [44], but has not been used for complexes between E1-activating enzymes and UBLs. The co-crystal structure of the Uba4_C202K_-Urm1 complex also showed a homodimer of Uba4, but in comparison with the apo structure of Uba4, appears fully symmetric. As no distinct electron densities corresponding to the RHDs were observed, the binding of Urm1 seems to dissolve the dimer of RHDs and displace the RHD domains. The structure also reveals that the C-terminal ‘GG-motif’ of Urm1 occupies the groove normally engaged by the Uba4 linker in the apo structure. This displacement probably enhances the flexibility of the RHD dimer, facilitating the catalytically relevant rearrangement of the RHDs. In summary, the linker region is not only a simple connector between the AD and RHD, but it also actively contributes to the conformational flexibility required for substrate coordination and catalysis [26]. Finally, mutational analyses showed that the catalytic cysteine of Uba4 not only mediates thioester bond formation but also protects the E1 enzyme from an unwanted conjugation with the product of the reaction, namely thiocarboxylated Urm1. However, the detailed molecular mechanisms underlying this self-protective function are not fully understood yet.

**Figure 3:**
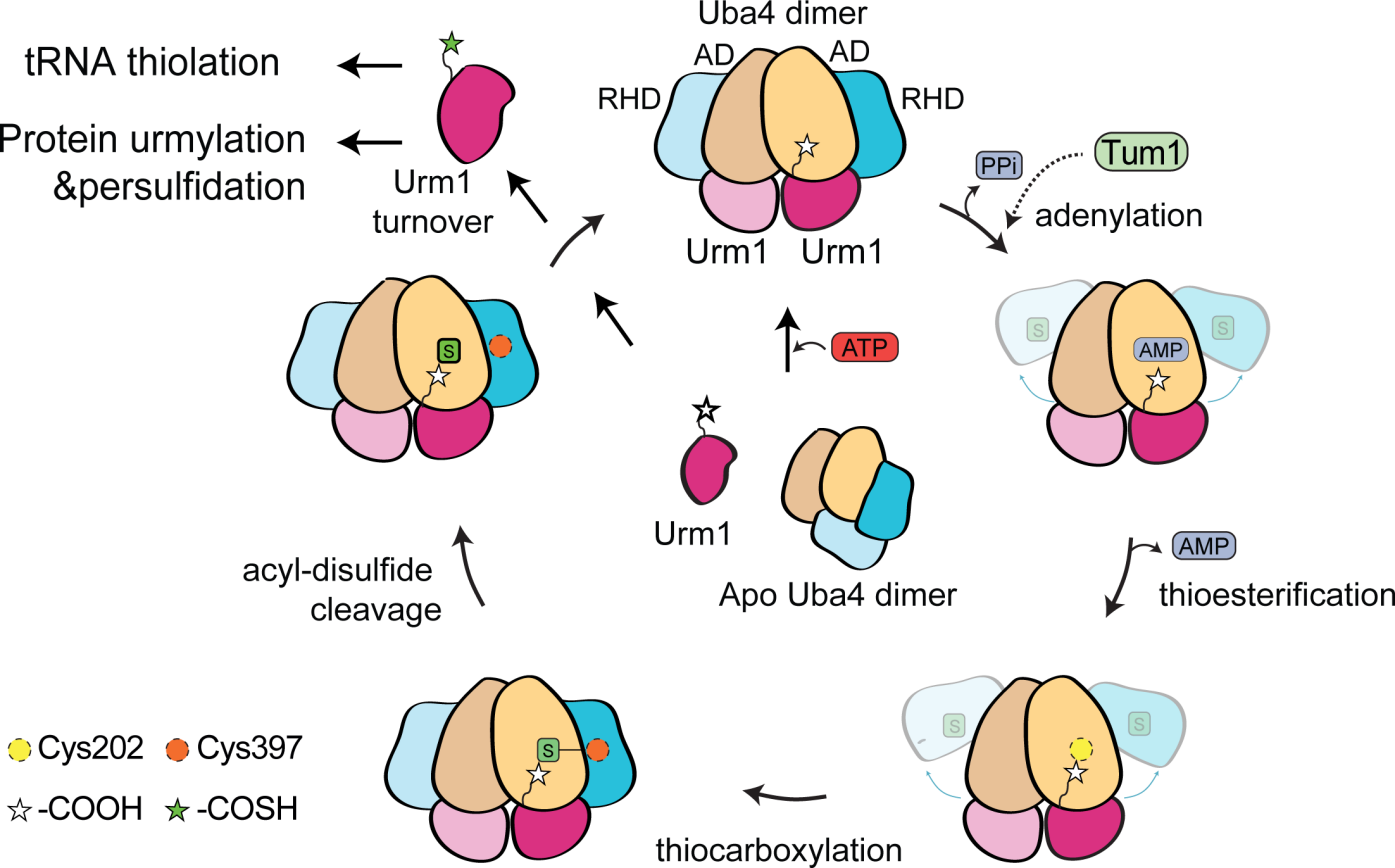
Schematic overview of the Urm1 thiocarboxylation reaction by Uba4.

To address the previously unresolved position of rhodanese-like domains (RHDs), the structure of the Uba4_C202K_-Urm1 complex was recently visualized using single particle cryo-electron microscopy (cryo-EM) [39]. The results of that study confirmed the previously proposed catalytic intermediates and provided new insights into the position of the RHD after their spatial rearrangement. In detail, each of the two RHDs was positioned in close proximity to the C-terminus of Urm1, suggesting that coordinated movements of the RHDs occur during the reaction cycle to facilitate the sulfur transfer from RHD to the C-terminus of Urm1 [39]. Disruption of the interface between the AD, RHD, and Urm1 impairs the sulfur transfer, underscoring the critical role of the newly identified domain interface in the Uba4-Urm1 complex. These structural observations also suggest that Urm1 and its C-terminus remain in almost identical relative position to the AD of Uba4 throughout adenylation, thioesterification, and thiocarboxylation steps until the release of thiocarboxylated Urm1 (Figure 3). Using mutational analyses, it was shown that the formation of thiocarboxylated Urm1 occurs even if the thioester intermediate cannot be formed between the conserved catalytic cysteine (Cys225) in the AD of Uba4 and adenylated Urm1 [26,39]. Persulfidation of Cys397 in the RHD alone was sufficient to directly transfer sulfur to the adenylated Urm1, indicating that the thioester intermediate promotes the catalytic reaction, but is not essential for product generation. Furthermore, Tum1 seems to provide a safeguard mechanism for the RHD, as this stand-alone sulfur-transferase can partially rescue the thiocarboxylation activity of the Uba4_C397A_ mutant [39], further supporting the functional plasticity of the thiolation cascade.

Upon Urm1 binding to the adenylation domain (AD) of Uba4, the dimeric rhodanese-like domains (RHDs) dissociate from the AD and move apart. The carboxylated C-terminus of Urm1 (COOH, white star) is first adenylated by the AD, and a transient thioester intermediate can form with a conserved cysteine in the AD (Cys202). Tum1 serves as a sulfur donor or alternatively, it may functionally replace the RHD domain. The persulfidated cysteine in the RHD (Cys397) attacks the C-terminal carbonyl of Urm1, forming an acyl-disulfide intermediate. This intermediate is resolved through a reductive cleavage step, likely involving a second yet uncharacterized cysteine. After the release of Urm1 with a thiocarboxylated C-terminus (COSH, green star) a new cycle begins with the recruitment of a fresh Urm1 molecule. The released Urm1-SH acts in tRNA thiolation or urmylation and persulfidation of target proteins.

In summary, the recent studies have provided deep molecular insights into the individual steps of the reaction cycle of Uba4 that uses ATP to relay an activated sulfur group onto the C-terminus of Urm1, needed for the downstream thiolation of tRNAs, urmylation, and the persulfidation of cysteine residues in target proteins.

### A unique E2/E3-independent UBL conjugation pathway

**Figure 4:**
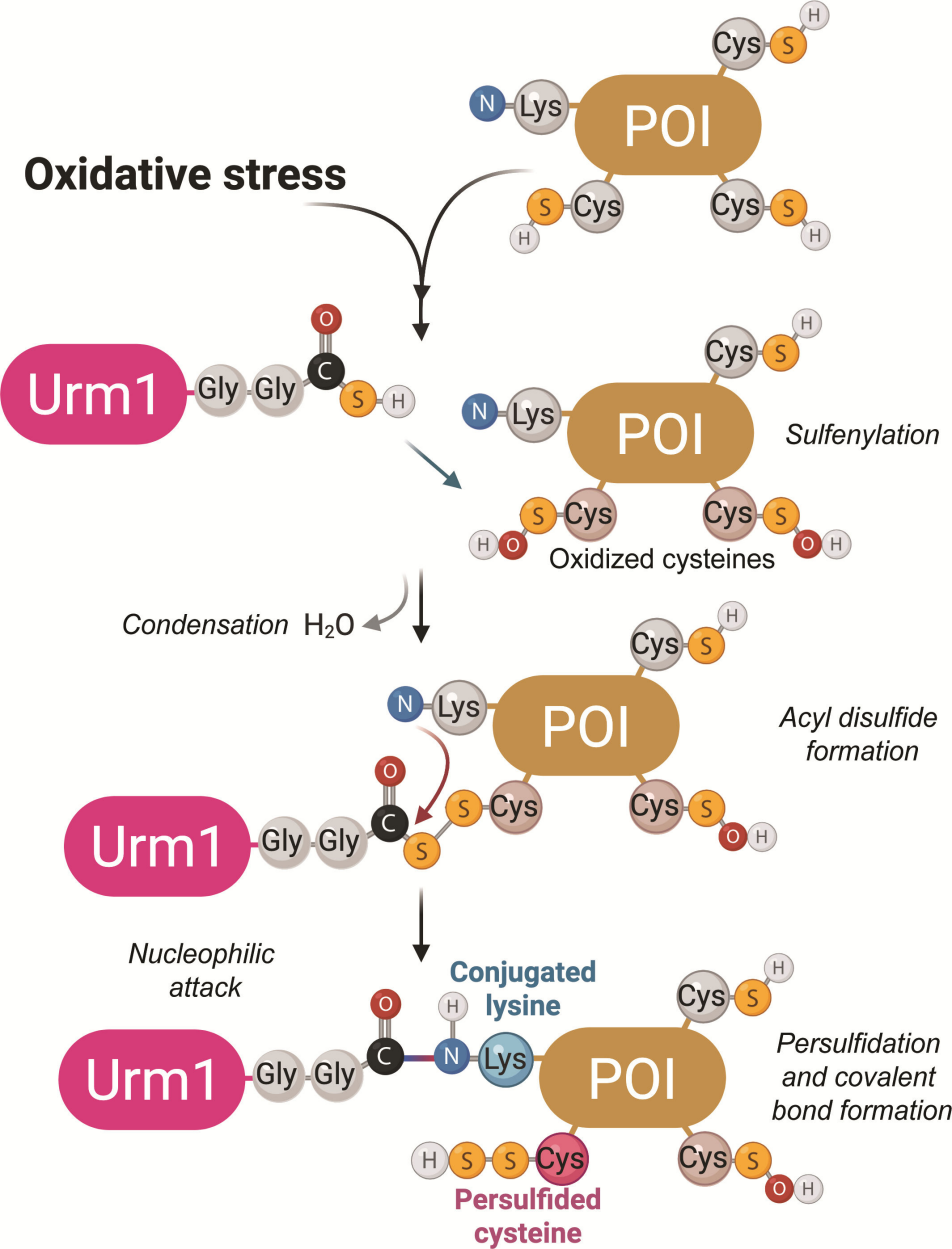
Mechanism of Urm1 conjugation and cysteine persulfidation.

Urm1 is an atypical ubiquitin-like modifier that seems to get activated under oxidative stress conditions, when redox-active cysteines in target proteins become sulfenylated. Once Uba4 has generated thiocarboxylated Urm1, the activated UBL can react with those cysteines to form a transient acyl-disulfide intermediate. Subsequently, a nearby nucleophilic residue, typically a lysine (or alternatively a serine or a threonine), can attack this intermediate, resulting in a covalent conjugation reaction that simultaneously triggers persulfidation of the cysteine residue (Figure 4).

The urmylation reaction mechanism effectively integrates ubiquitin-like conjugation with the sulfur transfer observed in the target protein. Specifically, the exposure of a redox-active cysteine (Cys, yellow) within the target protein to oxidative stress results in the sulfenylation of its side chain (‐SOH). Urm1‐SH identifies the sulfenylated cysteine, and subsequent condensation leads to the formation of a short-lived acyl disulfide intermediate. The reduction of this transient acyl disulfide intermediate can facilitate the recycling of Urm1‐SH. Alternatively, a nearby lysine (Lys, blue), serine, or threonine residue in proximity to the linked Cys‐Cys pair can initiate a nucleophilic attack on the acyl disulfide. This reaction pathway concurrently generates a persulfidated cysteine and establishes a covalent conjugation site between the C-terminus of Urm1 and the corresponding lysine (Lys, blue), serine, or threonine residue in the target protein. Note that only lysine linkage is depicted in the figure. Illustration created with Biorender.com.

The co-crystal of an ‘urmylated’ target protein revealed that Urm1 forms an isopeptide bond with Lys63 of Ahp1, while complementary mass spectrometry analyses have shown that multiple lysine residues (or serine and threonine residues) can serve as conjugation sites for Urm1 [9]. Spatial proximity to a redox-active cysteine rather than sequence specificity seems to be the key requirement for this targeted conjugation-coupled modification reaction. This rather flexible mechanism allows Urm1 to conjugate efficiently, even in the absence of typical lysine acceptors, highlighting its structural adaptability and broad range of potential targets.

****The conjugation of Urm1 is uniquely coupled to the sulfur transfer that results in cysteine persulfidation, which is a reversible and protective post-translational modification [45–47]. Persulfidation converts thiol (-SH) groups from sulfenic acids (SOH) on cysteines into persulfides (-SSH), which are more resistant to irreversible overoxidation (e.g., to sulfinic or sulfonic acids). This process preserves protein function under oxidative stress by maintaining the redox activity of key cysteine residues [45–47]. Urm1-mediated persulfidation has been observed in several proteins *in vitro*, including GAPDH, a central metabolic enzyme that undergoes redox regulation [48]. Persulfidation of GAPDH’s catalytic cysteine not only shields it from oxidative inactivation but also enhances its enzymatic activity, underscoring the physiological relevance of this modification [48].

The protective role of persulfidation extends to various proteins involved in detoxification, metabolism, and signaling [17,45,47,49]. Mechanistically, this is tightly linked to an oxidative environment, where sulfenylated cysteines act as electrophilic traps for Urm1-SH, enabling site-specific modification. Persulfidation is a dynamic and reversible process. Cellular redox systems, such as the thioredoxin and glutaredoxin pathways, can reduce persulfidated cysteines to their original thiol state, allowing the reversible modulation of protein activity [47]. The metabolic regulation of this modification is further achieved by its dependency on the intracellular levels of reactive sulfur species, like hydrogen sulfide (H₂S), which act as upstream mediators of persulfidation. In summary, the recent discovery of the link between Urm1 and persulfidation has revealed a novel targeted redox-protection mechanism that is not only conserved but also functionally embedded in cellular stress responses and metabolic regulation [49,50].

### Open questions and future research

Research on Urm1 continuously opens critical avenues for understanding translation, tRNA biology, redox biology, post-translational protein modification, and the cellular responses to stress. One of the key questions is related to the existence of a potential ‘deurmylase’, which in similarity to other known deubiquitinase (DUBs) would actively remove attached Urm1 from target proteins. The activity of such yet unidentified Urm1-specific DUB would clarify discrepancies in Urm1 target profiles across organisms and provide insights into the dynamic regulation of the Urm1 conjugation patterns. The role of Urm1 in stress sensing is particularly intriguing. Urm1 facilitates stress-dependent condensate formation and oxidative-stress-dependent cysteine persulfidation. It remains to be shown whether these two mechanisms are functionally related, by analyzing persulfidation levels of condensates. Further investigations are needed to define the ultimate list of Urm1 targets and the balance between covalent modification and noncovalent condensation. In addition, the protective function of Urm1 might also be therapeutically exploited to combat cellular aging, protect cellular proteomes, and reverse redox imbalances. Importantly, persulfidation is linked to ferroptosis, a form of regulated cell death driven by iron-dependent lipid peroxidation [17,51,52]. By scavenging free radicals and preventing thiol oxidation, persulfides promoted by Urm1 can mitigate lipid peroxidation and reduce ferroptotic susceptibility. These mechanisms may be exploited to develop novel therapeutic strategies for diseases characterized by oxidative damage, such as neurodegeneration or specific forms of cancer. Even if stress signaling seems to be the key trigger to activate the conjugation of Urm1, it remains elusive how this increased activity affects the role of Urm1 in tRNA thiolation. In detail, it is unclear whether the pool of available thiocarboxylated Urm1 is large enough to support both routes simultaneously or whether its dosage regulation is a relevant regulatory mechanism to reduce translation during stress. Furthermore, the molecular details of how thiocarboxylated Urm1 interacts with the Ncs2/Ncs6 complex during the specific tRNA thiolation reaction remain unknown. Finally, technological challenges related to the small size, the unavailable C-terminus, or problems with detecting untagged Urm1 in cells need to be addressed in the near future. Moreover, elucidating the evolutionary significance of the Urm1-Uba4 system as a molecular bridge between prokaryotic SCPs and eukaryotic UBLs remains a compelling area of research.

In summary, Urm1’s dual roles in protein conjugation and redox regulation underscore its biological importance and its unique role as a cellular protector with a significant therapeutic potential.

## Open Access

This article has been published open access under our Subscribe to Open programme, made possible through the support of our subscribing institutions, learn more here: https://portlandpress.com/pages/open_access_options_and_prices#conditional

## Abbreviations

RHD: rhodanese
SCPs: sulfur carrier proteins
UBL: ubiquitin-like
Uba4: ubiquitin-like protein activator 4
